# Can we really treat mentally ill patients involuntarily?

**DOI:** 10.1192/j.eurpsy.2022.922

**Published:** 2022-09-01

**Authors:** J. Gonçalves Cerejeira, G. Medina Ojeda, I. Santos Carrasco, C. De Andrés Lobo, T. Jiménez Aparicio, C. Vallecillo Adame, G. Guerra Valera, M. Queipo De Llano De La Viuda, A. Gonzaga Ramírez

**Affiliations:** 1 Hospital Clínico Universitario de Valladolid, Psychiatry, Valladolid, Spain; 2 Clinical Hospital of Valladolid, Psychiatry, Valladolid, Spain; 3 Hospital Clínico Universitario de Valladolid, Psiquiatría, VALLADOLID, Spain; 4 Hospital Clínico Universitario, Psiquiatría, Valladolid, Spain

**Keywords:** involuntary treatment

## Abstract

**Introduction:**

The therapeutic alliance is critical to the efficacy of psychiatric treatment and can be weakened by involuntary treatment measures. In Western culture, mental illness is still associated with violence and if significant risk of violence is detected, in Spain a civil court can order the application of involuntary treatments such as Involuntary Outpatient Commitment.

**Objectives:**

To discuss the effectiveness of some psychiatric involuntary treatments used in Spain.

**Methods:**

- Literature review about involuntary psychiatric treatments used in Spain - Case report about a patient undergoing Involuntary Outpatient Commitment

**Results:**

We present the case of a 54-year-old man, diagnosed with schizophrenia, admitted to our acute psychiatric yard more than five times due to violent behavior and psychotic symptoms. Five years ago, he was summitted to a period of three years of Involuntary Outpatient Commitment. In Spain this measure can include the administration of involuntary medication, an injectable antipsychotic treatment in this case. At the end of the order, he immediately stops attending consultations and abandoned psychopharmacological treatment.

**Conclusions:**

Involuntary Outpatient Commitment is a controversial measure and it stirs up the concepts of stigma, coercion, care, patient autonomy and, globally, the values of humanization in psychiatry.

**Disclosure:**

No significant relationships.

